# Optimization of carvedilol solid lipid nanoparticles: An approach to control the release and enhance the oral bioavailability on rabbits

**DOI:** 10.1371/journal.pone.0203405

**Published:** 2018-08-30

**Authors:** Khalid Mohamed El-Say, Khaled Mohamed Hosny

**Affiliations:** 1 Department of Pharmaceutics, Faculty of Pharmacy, King Abdulaziz University, Jeddah, Saudi Arabia; 2 Department of Pharmaceutics and Industrial Pharmacy, Faculty of Pharmacy, Beni-Suef University, Beni-Suef, Egypt; University of South Alabama Mitchell Cancer Institute, UNITED STATES

## Abstract

Solid lipid nanoparticles (SLNs) are prospective carriers for oral delivery of poorly soluble drugs with low bioavailability. Therefore, the study aimed at developing carvedilol (CVD) in SLNs to control its release and enhance its bioavailability in the management of hypertension, and cardiac diseases. Box-Behnken design (BBD) was applied to optimize the variables affecting the quality of CVD-SLNs which prepared by homogenization-ultrasonication technique. The concentrations of Percirol (X_1_), Gelucire (X_2_), and stearylamine (X_3_) were chosen as the crucial independent variables. The dependent variables were estimated and analyzed by Statgraphics software to achieve the optimum characteristics of the developed SLNs_._ The optimized SLNs was evaluated *in vitro* and *in vivo* for pharmacokinetic parameters on male New Zealand white rabbits. The results of this study revealed that the CVD-SLNs have a colloidal size of 31.3 nm with zeta potential of 24.25 mV indicating good stability and 91.43% entrapment efficiency. The *in vitro* release of CVD from the SLNs was best fitted to Hixon-Crowell model that describes the release from the particles with uniform size. The *in vivo* pharmacokinetics results indicated the prolongation in the mean residence time of CVD to 23 h when delivered in SLNs and its oral bioavailability enhanced by more than 2-folds.

## 1. Introduction

Oral drug delivery especially in a nano-size range has engrossed substantial pharmaceutical concern. In the middle of 1990s, solid lipid nanoparticles (SLNs) were utilized as a nano-carrier system for oral drug delivery [1]. It was reported that the oral administration of drugs in a nano-particulate system reduced the erratic absorption and subsequently increased the bioavailability owing to the adhesive properties of nanoparticles to the intestinal mucosa [2]. As a result of their submicron-size, SLNs were absorbed through intestinal mucosa either by paracellular pathway or by the intracellular uptake.

Carvedilol (CVD) encounters first-pass metabolism after oral administration that lead to its low bioavailability (about 25%) with a short elimination t_1/2_ of 2 h [3,4]. The aqueous solubility of CVD is about 100 μg/ml at pH 5 and this solubility decreased with increasing the pH which explain its low availability at the absorptive site [5]. So, the loading of CVD in SLNs enhances its intestinal absorption and consequently improves its oral bioavailability.

SLNs were considered as a substitute delivery system to other lipid based and polymeric systems as it pools their benefits like biocompatibility, biodegradability, controlling the drug release, and protecting the active drugs [6,7]. In addition, numerous methods can be used for preparation of SLNs such as the solvent emulsification-diffusion, solvent evaporation, microemulsion and double emulsion, high-pressure homogenization, and ultrasonication/ high speed homogenization method [8–11]. However, the homogenization technique is more advantageous than the other due to its production time is short, the homogenization lines are available in industry, the homogenization tools are acceptable by the regulatory authorities, the scale-up is easy, and the organic solvents are avoided in the production process [12,13]. Moreover, drugs incorporated in SLNs can be administered by several routes such as oral, parenteral, rectal, topical, and ophthalmic.[14–16]

An efficient and methodical tool in the design of drug delivery systems is the experimental design. Statistical designs allow a rational study of the effect of formulation parameters on the selected responses [17]. Development of drug delivery through the traditional procedure is a time, money and energy consuming approach as it based on the changing of one variable at a time while keeping other variables constant. Utilization of the design of experiment (DOE) technique permits investigating large number of factors simultaneously in few experimental runs [18–23].

So, the aim of the present study was to develop and optimize CVD-SLNs for efficient oral treatment of hypertension, and cardiac diseases. Furthermore, the wonderful aim of the research was to investigate statistically the effect of the factors on the characteristics of CVD-SLNs that control the release of CVD and enhance its oral bioavailability compared with the drug in a suspension.

## 2. Materials and methods

### 2.1. Materials

Carvedilol was kindly gifted by Riyadh Pharma, (Riyadh, Saudi Arabia). Precirol ATO5^®^ (glyceryl palmitostearate) and Gelucire 44/14^®^ (lauroyl macrogolglycerides) were kindly supplied by Gattefosse (Saint-Priest Cedex, France). Stearylamine was purchased from Fluka chemical company (Buchs, Switzerland).

### 2.2. Experimental design

Based on our previously published screening study of seven factors that influencing the homogenization/ ultra-sonication technique in preparation of SLNs, three factors have been scaled up for this optimization study [24]. BBD will be utilized to explore the influence of the three factors, Percirol ATO5 percentage (X_1_) as the solid lipid, Gelucire 44/14 percentage (X_2_) as the surfactant, and stearylamine percentage (X_3_) as the positive charge inducing agent in 15 batches. The optimization will be conducted to develop CVD-SLNs with minimum particle size (Y_1_), maximum of both zeta potential and entrapment efficiency (Y_2_ and Y_3_), and controlled release behavior (Y_4_ and Y_5_) using Statgraphics^®^ Centurion XV, Software, Version 15.2.05 (StatPoint, Inc., Warrenton, VA).

### 2.3. Preparation of carvedilol solid lipid nanoparticles

The formulations of CVD-SLNs based on BBD were prepared according to the hot homogenization followed by ultra-sonication technique as described previously [25,26]. Briefly, CVD was added to the molten lipid and stirred to be completely dissolved. Then add slowly the hot Gelucire 44/14 solution and stearylamine to the drug-lipid melt and then homogenized at 10,000 rpm for 3 min maintaining the temperature at 80°C (Ultra-turrax T- 25 IKA-Werke GmbH & Co. KG, Staufen, Germany). Further, the obtained emulsion was sonicated using the probe sonicator for 5 min and set aside at room temperature to cool (Sonics Vibra Cell, VCX 750, Sonics & Materials, Inc. (Newtown, CT). The control SLN formulations were prepared in similar manner without addition of CVD.

### 2.4. Determination of the particle size, polydispersity, and zeta potential

The average particle size (PS), polydispersity index (PDI), and zeta potential (ZP) of CVD-SLN batches were estimated by dynamic light scattering (DLS) technique using Zetatrac instrument (Microtrac, Inc., Montgomeryville, PA). The samples were diluted with distilled water and sonicated for 5 s to eradicate air bubbles and break up aggregates. The resultant colloidal dispersions were examined in triplicate.

### 2.5. Determination of the entrapment efficiency

The percent of CVD entrapped in the prepared SLNs was determined by an ultracentrifugation method as described before [27,28]. Briefly, the sample was centrifuged, filtered and the amount of free and entrapped CVD was dissolved in methanol and analyzed at 248 nm using the HPLC method [29]. Eq 1 was used for the calculation of the entrapment efficiency (EE %):

$$EE\left(\%\right)=\frac{T_{S}-F_{S}}{T_{S}}\times 100$$ (1)

where Fs is the amount of free CVD diffused in collection section after ultracentrifugation, and Ts is the calculated CVD concentration used in the formulation of SLNs. All measurements were performed in triplicate.

### 2.6. *In vitro* release study

The release of CVD from SLNs was performed using the dialysis bag method [30]. Briefly, the dialysis bags (MWCO of 12 kDa, Sigma Aldrich, USA) containing the CVD-SLNs were dipped in the baskets of the USP dissolution apparatus I. 500 ml of 0.1N HCl pH 1.2 was used as a dissolution medium at 37°C and the medium pH was slightly raised by addition of 0.05M phosphate buffer solution of pH 8.0 portion wise until pH 7.4 with constant stirring at 50 rpm. At the predetermined 12 time intervals over 12 h, aliquots of 5 ml were withdrawn and immediately replenished with the fresh medium. The samples were analyzed for CVD concentration using the HPLC method after suitable dilution [29].

### 2.7. Mathematical modelling of carvedilol release from solid lipid nanoparticles formulations

The release data were mathematically treated according to zero, first, second-order, Higushi diffusion, Hixon-Crowell cube root law, Baker-Lonsdale, and Korsmeyer–Peppas release kinetic models. The model with the highest correlation coefficient was considered to be the best model and the values of the exponent (n) were used for determining the most fitting model to explain the release mechanism.

### 2.8. Prediction, preparation, and evaluation of the optimized formulation

Using the Statgraphics software, the obtained data for each response were analyzed and after the multiple response optimization, the optimized CVD-SLNs formulation was predicted, prepared and evaluated for all responses (Y_1_-Y_5_). Moreover, the optimized formulation was scaled up for the *in vivo* study.

### 2.9. *In vivo* and pharmacokinetic study on rabbits

#### 2.9.1. Subject population

The *in vivo* study was carried out on male New Zealand white rabbits (2.5 ± 0.17 kg) on administration of 1 mg/kg single oral dose of CVD. The *in vivo* study protocol was revised and approved by the Animal Ethics Committee, Faculty of Pharmacy, King Abdulaziz University (Approval No. 1061439). The study fulfilled with the Declaration of Helsinki, the Guiding Principle in Care and Use of Animals (DHEW production NIH 80–23), and the "Standards of Laboratory Animal Care" (NIH distribution #85–23, reconsidered in 1985). The rabbits were fasted for one day preceding to the study and were allowed free access to water. The rabbits were divided into two groups (6 per group) those administered CVD orally with the same dose. The first group administered the optimized CVD-SLNs dispersed in distilled water (Test) and the other received CVD suspended in distilled water (Reference). 0.25% sodium carboxy methyl cellulose was used to suspend CVD in distilled water for enough time to give the dose. The study was conducted using a comparative, randomized single-dose, open-label, parallel study design.

#### 2.9.2. Sample collection and chromatographic analysis

The blood samples (0.5 ml) were withdrawn from the marginal ear vein at predetermined time points (0, 0.5, 1, 2, 4, 6, 8, 12, and 24 h) after oral administration of the test/reference formulations. Afterward, the samples were centrifuged at 3,500 rpm for 5 min and then the obtained plasma samples were stored at −20°C until assay using the previously reported modified HPLC method [29,31].

#### 2.9.3. Pharmacokinetic analysis

Non-compartmental pharmacokinetic analysis for CVD plasma concentrations was done in order to estimate the different parameters controlling the pharmacokinetic of the drug in the tested plasma samples. Maximum plasma carvedilol level and time to reach this level were determined for both test and reference. Area under Plasma concentration time curve for both formulations were determined in order to estimate the relative bioavailability of the optimized CVD-SLNs formula compared to the reference. Mean residence time were determined to ensure the controlled release manner for CVD when administered as CVD-SLNs. The elimination rate constant, elimination half-life, and total body clearance were measured to indicate if the administration of CVD as SLNs whether or not cause prolongation in its duration of action. The data were expressed as the mean ± standard deviation and analyzed with Kinetica® software (Version 4, Thermo Electron Corp., MA, USA).

#### 2.9.4. Statistical analysis of the data

The data was analyzed with GraphPad Prism 6 (GraphPad Software, San Diego, CA). Two-way analysis of variance (ANOVA) followed by Tukey’s multiple comparisons test was used to assess the significance of the difference between the optimized CVD-SLNs and the drug in suspension.

## 3. Results and discussion

In this study, 15 formulations of CVD-SLNs were prepared as suggested by BBD. Incorporation of CVD in SLNs as a drug carrier to achieve the controlled drug release behavior, and enhance the oral bioavailability of entrapped CVD.

### 3.1. Evaluation of the prepared CVD-SLNs

The particle size of the prepared formulations did not exceed 100 nm and ranged from 20 ± 0.09 nm in F5 to 58 ± 2.09 nm in F6 with PDI of not more than 0.3. This finding reflected the narrow particle size distribution with unimodal pattern of NPs as displayed in Table 1. Surface modification of NPs with positively charged stearylamine (SA) increase the zeta potential to 25.2 ± 1.33 mV which indicate the acceptable stability of the colloidal dispersion. In addition, the prepared SLNs entrapped CVD with high efficiency that was reached to 95 ± 4.12% in F7.

**Table 1:** Design matrix including investigated factors with their levels and the observed values of responses (Y_1_- Y_5_) for 15 formulations of carvedilol-loaded solid lipid nanoparticles.

Batch No.	Factors			Responses *				
	X_1_	X_2_	X_3_	Y_1_ (nm)	Y_2_ (mV)	Y_3_ (%)	Y_4_ (%)	Y_5_ (h)
1	12	3	5	27 ± 0.74	15.6 ± 0.35	92 ± 5.74	19.0 ± 1.15	8.50 ± 0.19
2	8	2	6	31 ± 0.34	22.8 ± 0.52	80 ± 4.21	16.0 ± 0.62	9.25 ± 0.21
3	8	2	4	35 ± 0.65	7.2 ± 0.26	79 ± 2.24	18.0 ± 0.86	9.00 ± 0.38
4	10	3	6	23 ± 0.15	24.4 ± 0.91	83 ± 4.42	23.0 ± 1.15	8.00 ± 0.26
5	8	3	5	20 ± 0.09	13.7 ± 0.65	78 ± 5.17	28.0 ± 1.07	7.25 ±0.09
6	12	1	5	58 ± 2.09	15.1 ± 0.82	93 ± 6.34	5.0 ± 0.15	11.25 ± 0.42
7	12	2	6	44 ± 0.34	21.7 ± 1.13	95 ± 4.12	7.0 ± 0.33	10.50 ± 0.36
8	10	3	4	24 ± 1.25	5.3 ± 0.13	83 ± 1.25	25.0 ± 1.21	7.75 ± 0.53
9	10	1	4	53 ± 0.39	8.4 ± 0.55	84 ± 5.33	8.0 ± 0.31	10.25 ± 0.61
10	10	1	6	50 ± 0.84	25.2 ± 1.33	85 ± 6.64	6.0 ± 0.25	11.0 ± 0.79
11	12	2	4	47 ± 1.37	6.1 ± 0.39	94 ± 3.71	9.0 ± 0.45	10.25±0.49
12	8	1	5	49 ± 1.12	14.2 ± 0.93	79 ± 1.45	13.0 ± 0.75	9.50 ± 0.39
13	10	2	5	38 ± 0.47	12.5 ± 0.75	86 ± 2.54	10.0 ± 0.61	10.0 ± 0.68
14	10	2	5	41 ± 0.54	12.6 ± 0.82	84 ± 3.75	10.4 ± 0.55	9.90 ± 0.35
15	10	2	5	39 ± 0.26	12.8 ± 1.05	83 ± 5.12	10.3 ± 0.45	10.1 ± 0.54

Notes:

*Data are expressed as the mean ± standard deviation (n = 3).

**Abbreviations:** Percirol ATO5 concentration, X_1_; Gelucire 44/14 concentration, X_2_; Stearylamine concentration, X_3_; Particle size, Y_1_; Zeta potential, Y_2_; Entrapment efficiency, Y_3_; Amount of drug release after 1h, Y_4_; Time for 85% release, Y_5_.

### 3.2. *In vitro* release studies

**Fig 1:**
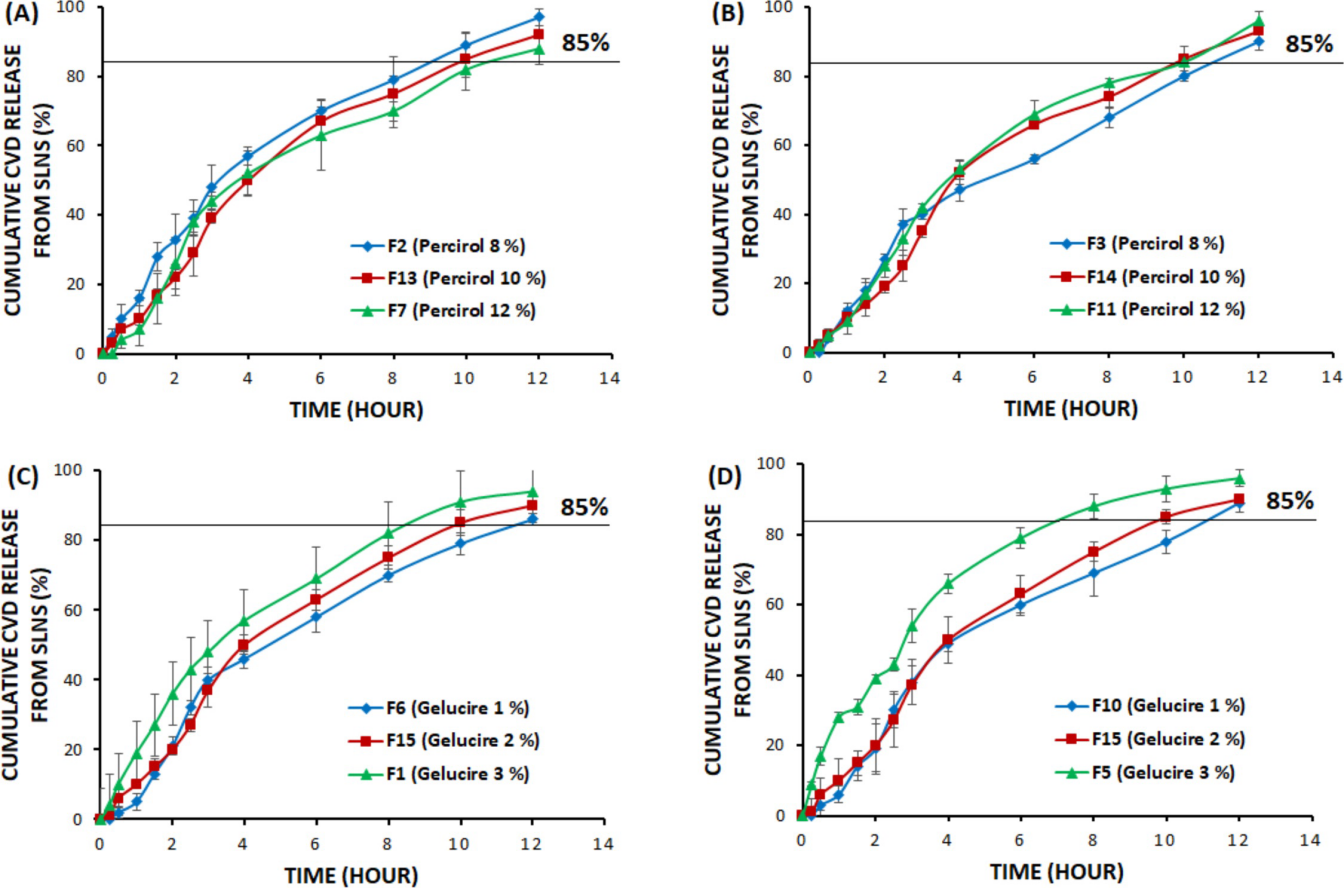
In vitro release profiles for CVD-SLN formulations with different concentrations of Percirol ATO5 and Gelucire 44/14. As a result of overlapping, error bars are omitted for clarity.

The release behavior of CVD from SLNs was studied using the dialysis bag method. The initial release percentage after 1 h (Y_4_) and the time required for 85% of drug release (Y_5_) to achieve the controlled behavior were calculated and presented in Table 1. The initial CVD release ranged from 6% as displayed in F10 to 28% as displayed in F5. Whereas, the cumulative CVD release after 12 h ranged from 89% to 98% in F10 and F8, respectively. Moreover, the time required for 85% release varied in all formulations and reached 11.25 h as in F6 which confirm the controlled release behavior of CVD from SLNs. It was found that the release was controlled as the pH of the medium increased which in accordance with the reported finding [32]. It was observed that the release of CVD from SLNs can be influenced by both Percirol and Gelucire concentration as depicted in the previous studies [24,32]. (Fig 1A and 1B) shows the inverse correlation between the Percirol content in the nanoparticles and the CVD release. As the percent of Percirol increased the release percentage will decreased which may be due to the higher partition coefficient and lipophilicity of CVD, this lipophilicity decreased the diffusion of the drug from the lipid matrix formed from Precirol to the aqueous dissolution medium and by increase the thickness and integrity of this lipid matrix by increase Precirol concentration, the diffusion occur with more difficulty and the release become more controlled and sustained [33]. In another words, as the Precirol concentration was increased, the release of CVD decreased which can be explained by increasing the efficiency of encapsulation of drug in the nanoparticles and increasing the thickness of the lipid coating consequently reducing drug partition in the outer phase which is in agreement with previous findings [34,35]. Conversely, (Fig 1C and 1D) shows the direct correlation between the Gelucire content in the nanoparticles and the CVD release. When the percent of Gelucire increased the release percentage will increased which can be explained by the higher hydrophilic lipophilic balance of Gelucire 44/14 (HLB = 11), which make it a suitable water dispersible surfactant for lipid based formulation and lipid soluble drugs through the enhancement of the wettability and dissolution of the lipophilic drugs. And by increase its concentration in the formulation, the percentage of the drug released was increased [36]. In addition, the surface activity of Gelucire 44/14 reduces the interfacial tension between the SLNs and the dissolution medium and minimizes the aggregation of drug particles and boosts the dissolution rate of CVD [37].

### 3.3. Kinetic treatment of CVD release from SLN formulations

The release data were treated with different kinetic orders and models (zero, first, or second order and Higuchi, Hixon-Crowell cube root law, and Baker-Lonsdale models) by comparing the correlation coefficients’ values to select the best fitted release one. Also, Korsmeyer-Peppas model was applied to determine the mechanism of CVD release from the SLN formulations.

The results of the in vitro release study on all formulations except F2 and F5 revealed that CVD release from nanoparticles were best fitted to Hixon-Crowell model, as revealed by the highest correlation coefficient (r) values. Hixon-Crowell model usually describes the release from the particles with uniform size and when the drug release occur by dissolution with change in surface area and diameter of the particles [38]. As mentioned earlier, all CVD-SLN formulations displayed the uniformity in their size and this making the drug release depend on the dimensional erosion of SLN itself which occur gradually not occur through rupture as in case of nanospheres. Fitting the data to Korsmeyer-Peppas model revealed that most of the SLN formulations showed the diffusional release exponent values (n = 0.5–1) which indicates the non-Fickian release mechanism that is controlled by a combination of diffusion and nanoparticle erosion [39,40].

### 3.4. Optimization of CVD-SLNs using response surface methodology

#### 3.4.1. Evaluation of the quantitative effects of the factors

**Fig 2:**
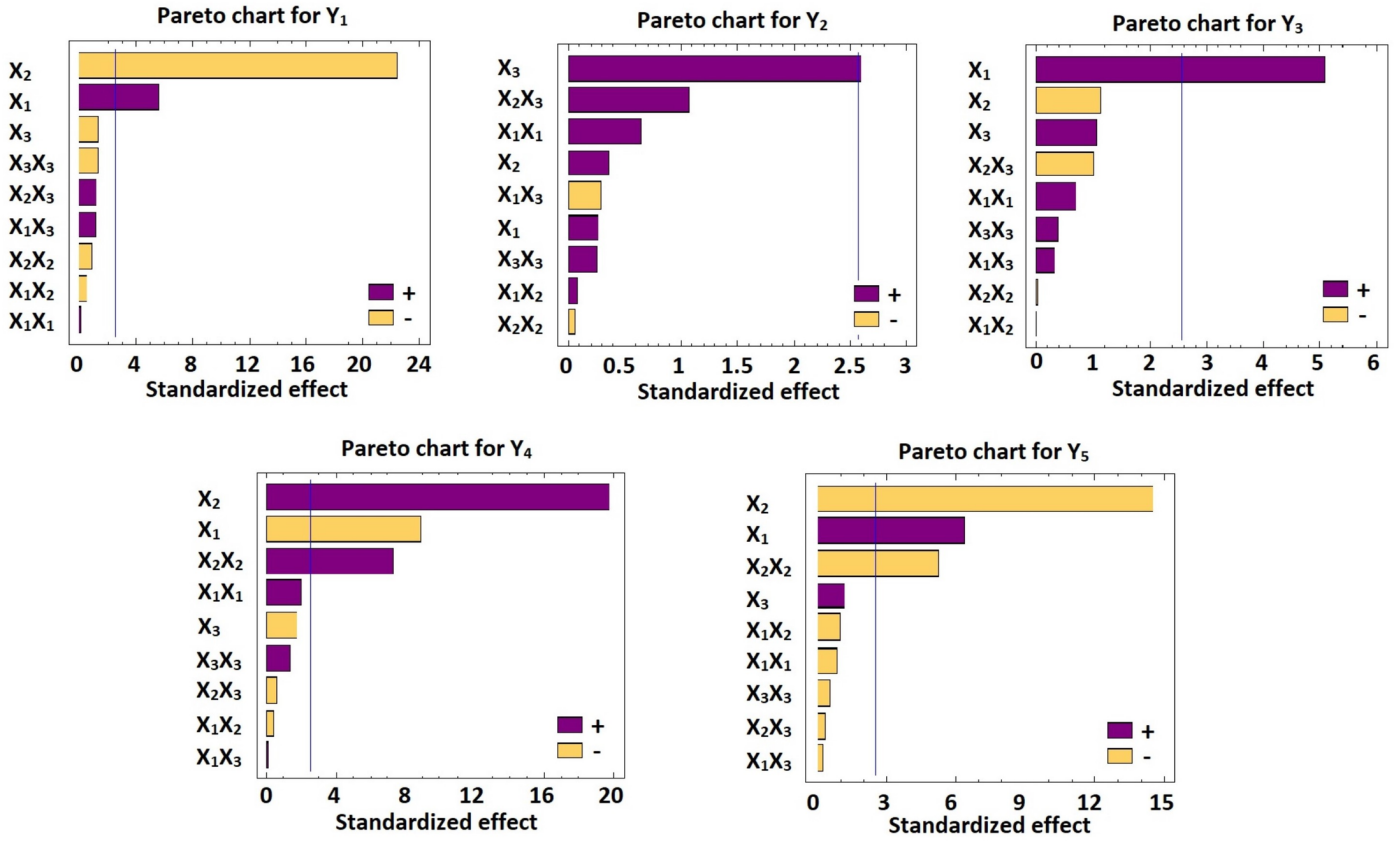
Standardized Pareto charts for Y_1_-Y_5_. Abbreviations: X_1_, Percirol ATO5 concentration; X_2_, Gelucire 44/14 concentration; X_3_, Stearylamine concentration; T85%, time for 85% of drug release; X_1_X_2_, X_1_X_3_ and X_2_X_3_ are the interaction terms between the factors; X_1_X_1_, X_2_X_2_ and X_3_X_3_ are the quadratic terms of the factors.

**Fig 3:**
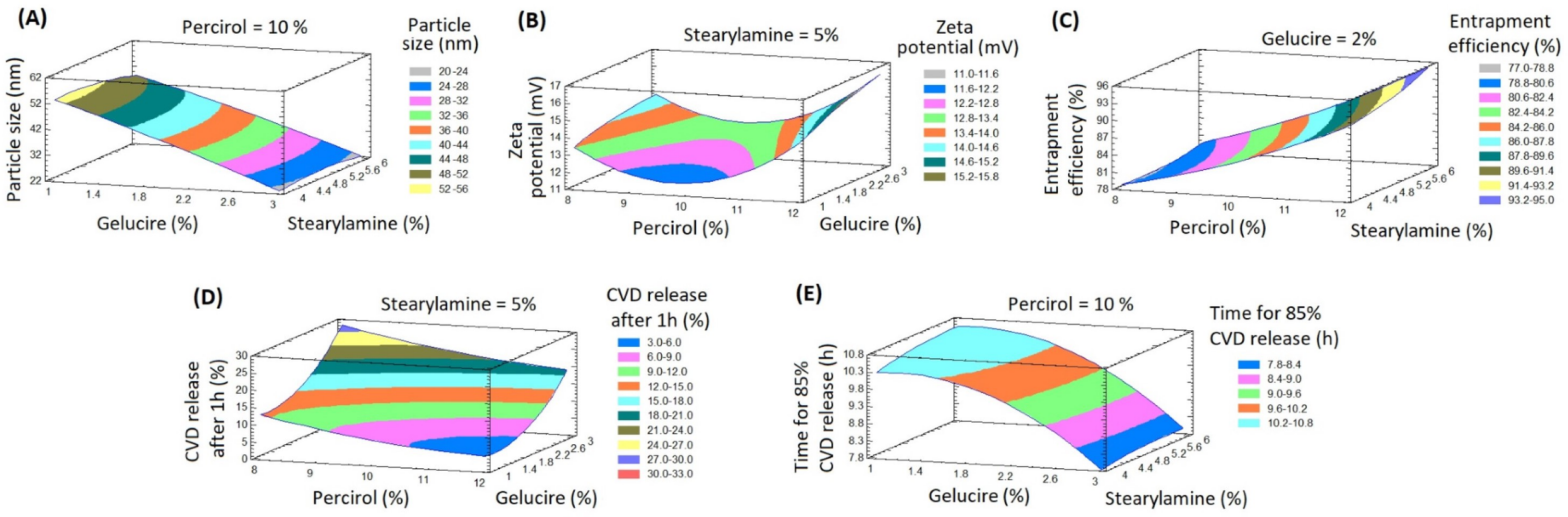
Estimated response surfaces with contour plots showing the effects of X_1_, X_2_, and X_3_ on the dependent variables (Y_1_–Y_5_).

ANOVA with multiple regression analysis of the responses (Y_1_- Y_5_) using Statgraphics software were implemented for statistical analysis of BBD formulations. The estimated factors effects with *p-values* on the five responses were presented in Table 2. The correlations between the investigated factors and the responses were presented in Pareto charts (Fig 2). Whereas, the effect of these factors on the responses were displayed in 3D response surface plots (Fig 3). It was found that Gelucire concentration (X_2_) and Percirol concentration (X_1_) showed significant effects on Y_1_ with *p-*values of 0.0001 and 0.0024, respectively. On the contrary, stearylamine concentration (X_3_) is the only factor that affects significantly the zeta potential of the nanoparticles dispersion (Y_2_) with *p-*value of 0.0484. Also, it was noticed that X_1_ displayed a significant synergistic effect on the entrapment efficiency (Y_3_) with *p-*value of 0.0038. The initial release after 1 h (Y_4_) is affected significantly with X_1_ and X_2_ with *p-*values of 0.0003 and 0.0001, respectively. While, the time for 85% drug release (Y_5_) is affected significantly with X_1_ and X_2_ with *p-*values 0.0014 and 0.0001, respectively. Also, it was noticed that the quadratic term of X_2_ significantly affects both Y_4_ and Y_5_ with *p-*values of 0.0007 and 0.0034, respectively.

**Table 2:** Statistical analysis of variance (ANOVA) of the responses (Y_1_- Y_5_) results.

Factors	Particle size (Y_1_)		Zeta potential (Y_2_)		Entrapment efficiency (Y_3_)		Initial release percentage after 1 h (Y_4_)		T85% release (Y_5_)	
	*Estimate*	*P-Value*	*Estimate*	*P-Value*	*Estimate*	*P-Value*	*Estimate*	*P-Value*	*Estimate*	*P-Value*
X_1_	9.017	0.0024*	1.4293	0.7968	13.758	0.0038*	-8.7931	0.0003*	1.4181	0.0014*
X_2_	-28.465	0.0001*	1.5086	0.7323	-2.482	0.2994	15.413	0.0001*	-2.53879	0.0001*
X_3_	-2.0517	0.2352	13.012	0.0484*	2.7241	0.3390	-1.6206	0.1448	0.24569	0.2956
X_1_X_1_	0.21839	0.9204	4.4046	0.5484	2.4425	0.5190	2.63736	0.0949	-0.24569	0.4319
X_1_X_2_	-1.0	0.6129	0.5	0.9380	0.0	1.0000	-0.5	0.6805	-0.25	0.3747
X_1_X_3_	2.96552	0.3058	-2.558	0.7772	1.4827	0.7502	0.08620	0.9592	-0.08620	0.8201
X_2_X_2_	-1.8850	0.4060	-0.371	0.9589	-0.109	0.9765	9.39598	0.0007*	-1.50431	0.0034*
X_2_X_3_	2.06897	0.2834	6.1172	0.3302	-2.965	0.3563	-0.6724	0.5548	-0.07758	0.7579
X_3_X_3_	-2.8505	0.2393	1.7873	0.8095	1.4080	0.7132	1.80977	0.2280	-0.16810	0.5939

Note:

* Significant effect of factors on individual responses.

**Abbreviations:** X_1_, Percirol ATO5 concentration; X_2_, Gelucire 44/14 concentration; X_3_, Stearylamine concentration; T85%, time for 85% of drug release; X_1_X_2_, X_1_X_3_, and X_2_X_3_ are the interaction terms between the factors; X_1_X_1_, X_2_X_2_ and X_3_X_3_ are the quadratic terms between the factors.

#### 3.4.2. Mathematical modelling of the experimental data

Depending on the analysis of the observed values of the responses; a mathematical model for each response was generated and presented in Eqs 2–6.

$$\text{Particle size}\;\left({\text{Y}}_{1}\right)=56.126-1.499{\text{X}}_{1}-13.135{\text{X}}_{2}+3.745{\text{X}}_{3}+0.027{\text{X}}_{1}{}^{2}-0.25{\text{X}}_{1}{\text{X}}_{2}+0.741{\text{X}}_{1}{\text{X}}_{3}-0.943{\text{X}}_{2}{}^{2}+1.034{\text{X}}_{2}{\text{X}}_{3}-1.425{\text{X}}_{3}{}^{2}$$ (2)

$$\text{Zeta potential}\;\left({\text{Y}}_{2}\right)=52.782-7.706{\text{X}}_{1}-15.046{\text{X}}_{2}-2.151{\text{X}}_{3}+0.551{\text{X}}_{1}{}^{2}+0.125{\text{X}}_{1}{\text{X}}_{2}-0.6396{\text{X}}_{1}{\text{X}}_{3}-0.186{\text{X}}_{2}{}^{2}+3.059{\text{X}}_{2}{\text{X}}_{3}+0.894{\text{X}}_{3}{}^{2}$$ (3)

$$\text{Entrapment efficiency}\;\left({\text{Y}}_{3}\right)=97.2299-4.52{\text{X}}_{1}+6.391{\text{X}}_{2}-6.419{\text{X}}_{3}+0.305{\text{X}}_{1}{}^{2}+0.0{\text{X}}_{1}{\text{X}}_{2}+0.371{\text{X}}_{1}{\text{X}}_{3}-0.055{\text{X}}_{2}{}^{2}-1.481{\text{X}}_{2}{\text{X}}_{3}+0.704{\text{X}}_{3}{}^{2}$$ (4)

$$\text{Initial release percentage after 1 h}\;\left({\text{Y}}_{4}\right)=90.451-8.649{\text{X}}_{1}-8.154{\text{X}}_{2}-9.402{\text{X}}_{3}+0.329{\text{X}}_{1}{}^{2}-0.125{\text{X}}_{1}{\text{X}}_{2}+0.022{\text{X}}_{1}{\text{X}}_{3}+4.698{\text{X}}_{2}{}^{2}-0.336{\text{X}}_{2}{\text{X}}_{3}+0.905{\text{X}}_{3}{}^{2}$$ (5)

$$\text{Time for 85\% drug release}\;\left({\text{Y}}_{5}\right)=-2.517+1.202{\text{X}}_{1}+2.558{\text{X}}_{2}+1.256{\text{X}}_{3}-0.031{\text{X}}_{1}{}^{2}-0.063{\text{X}}_{1}{\text{X}}_{2}-0.022{\text{X}}_{1}{\text{X}}_{3}-0.752{\text{X}}_{2}{}^{2}-0.039{\text{X}}_{2}{\text{X}}_{3}-0.084{\text{X}}_{3}{}^{2}$$ (6)

Eqs 2–6 reveal the quantitative effects of the factors on the responses (Y_1_-Y_5_). Plotting of Pareto charts defines the effect of the examined factors effects on the responses (Fig 2). It was found that the bars of Percirol % (X_1_) and Gelucire % (X_2_) extend beyond the reference line in Pareto chart for Y_1_, which endorses their significant effects on the particle size of the nanoparticles. As shown in Table 2 and Fig 2 the Percirol percentage (X_1_) has a synergistic effect while the Gelucire percentage has an antagonistic effect on Y_1_. This finding can be explained by the possibility of the fusion of the surfactant molecules with the lipid matrix at lower melting temperature, this fusion permit the surfactant to display its action and divide the lipid matrix into small globules within the aqueous layer which in a good agreement with the previous reported postulations [15,41]. Similar effects of X_1_ and X_2_ on Y_5_ have been explored. On the other hand, X_1_ has an antagonistic effect while X_2_ has a synergistic effect on the initial release percentage after 1 h (Y_4_). Also, X_1_ and X_3_ displayed a synergistic effects on the entrapment efficiency (Y_3_), and the zeta potential (Y_2_), respectively.

However, the Percirol % (X_1_) and Gelucire % (X_2_) in the SLN formulations significantly affect all of the investigated release parameters in this study (Y_4_ and Y_5_). It was noted from Figs 2 and 3 that a direct relationship exists between X_2_ and Y_4_, which indicates that the Gelucire % (X_2_) governs the initial percentage of CVD released after 1 h (Y_4_). At the same concentrations of both X_1_ and X_3_, as X_2_ increased from 1 to 3%, Y_4_ increased from 5 in F6 to 19% in F1, from 13 in F12 to 28% in F5, and from 6 in F10 to 23% in F4. This finding can be explained by the fact that, Gelucire 44/14 is a suitable water dispersible surfactant for lipid soluble drugs as carvedilol, it increase the dissolution rate of the lipophilic drugs in aqueous media. So, by increase its concentration in the formulation, the percentage of the drug released and dissolute was increased [42].

Regarding the effect of X_1_ on the release profile of CVD from SLNs the results indicated an inverse relationship between X_1_ and Y_4_ which indicates that X_1_ contributes in the determination of the initial percentage of CVD released after 1 h (Y_4_). At the same concentrations of both X_2_ and X_3_, as X_1_ increased from 8 to 12%, Y_4_ decreased from 18 in F3 to 9% in F11, from 16% in F2 to 7% in F7, and from 28 in F5 to 19% in F1. On the contrary, it was found from Figs 2 and 3 that a direct relationship exists between X_1_ and Y_5_, which indicates that X_1_ determines the time for 85% CVD release (Y_5_). At the same concentrations of both X_2_ and X_3_, as X_1_ increased from 8 to 12%, Y_5_ increased from 5 in F6 to 19% in F1, from 13 in F12 to 28% in F5, and from 6 in F10 to 23% in F4. This observation can be attributed to the fact that precirol represent the lipid matrix of the SLNs, and by increase its concentration, the thickness, viscosity, and integrity of the lipid matrix increase, and thus increase the difficulty of the diffusion and release of the lipid soluble drugs from the SLNs [33].

The same finding was achieved regarding to the effect of X_2_ on Y_5_. Figs 2 and 3 exhibited that an inverse relationship exists between X_2_ and Y_5_. When the Gelucire % increased from 1 to 3% in the SLN formulation with the same levels of X_1_ and X_3_, Y_5_ decreased from 11.25 to 8.50 h in F6 and F1, respectively; from 9.50 to 7.25 h in F12 and F5, respectively; and from 10.25 to 7.75 h in F9 and F8, respectively. This result could be owing to the decrease in particle size as Gelucire concentration decrease this make the release of the drug from the SLNs matrix is easier as the diffusion length decreased. Also, this could be due to the surfactant action of Gelucire in enhancing the dissolution and wettability of lipid soluble drugs as carvedilol in aqueous dissolution media [42].

### 3.5. Prediction, preparation, and evaluation of the optimized CVD-SLNs formulation

After the analysis of the obtained data and generating the regression equations [Eqs 2–6] that correlate the investigated factors and the observed responses, the process of preparation of CVD-SLNs was optimized. The suggested optimized formulation composed of Percirol, Gelucire, and stearylamine in concentrations of 11.99, 2.87, and 6%, respectively. To validate these values, the optimized CVD-SLN formulation was prepared and evaluated. The observed responses of this formulation were 31.3 nm, 24.25 mV, 91.43%, 17.55%, and 8.92 h for Y_1_, Y_2_, Y_3_, Y_4_, and Y_5_, respectively which in a close agreement with the predicted values. This proved the feasibility of the optimization procedure using Box-Behnken design in developing a new CVD-SLN formulation with controlled release and enhanced oral bioavailability.

### 3.6. *In vivo* and pharmacokinetic evaluation on rabbits

**Fig 4:**
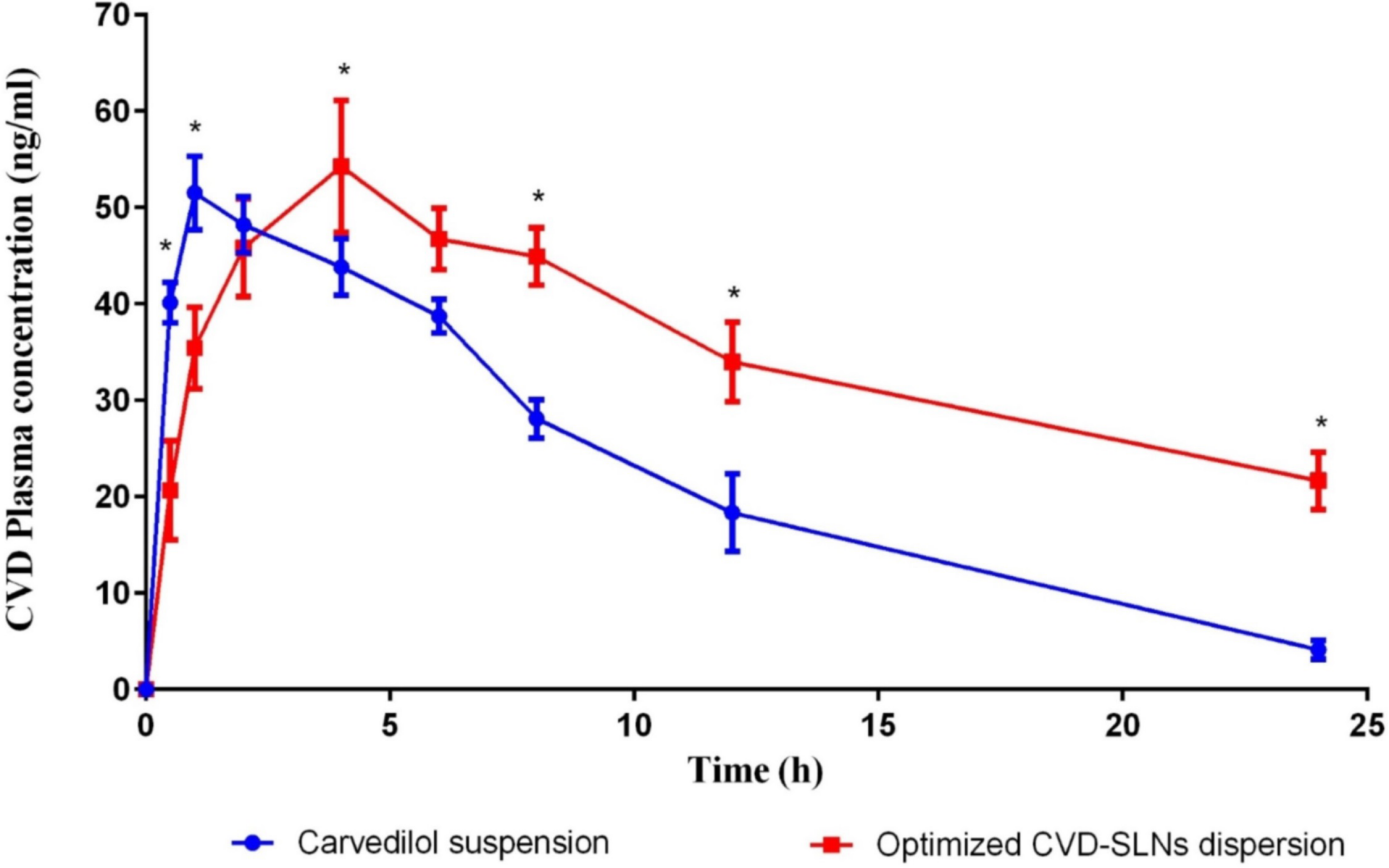
Mean plasma concentration-time profiles for CVD after oral administration of a single dose (1 mg/kg) of the CVD suspension and the CVD-SLNs formulations.

Fig 4 and Table 3 presented the mean plasma concentration-time profiles and the pharmacokinetic parameters of CVD after oral administration of a single dose of either the optimized CVD-SLNs formulation or the drug in suspension. The obtained pharmacokinetic parameters indicated the controlled and prolonged action of the drug and enhanced its oral bioavailability when delivered in SLNs. The half-life prolonged from 5.6 h in case of drug suspension to 15.3 h for SLNs, and the MRT also prolonged from 8.7 to 23.19 h. This prolongation in half-life and MRT proved the sustained and prolonged release and residence of the SLNs in blood stream. This could be due to the presence of Gelucire in the formulation of CVD-SLNs leads to a steric hindrance that decrease the tissue uptake by avoiding the reticuloendothelial system, this confirmed by the CVD clearance results which decreased from 0.0042 ml/h in case of drug suspension to 0.002 ml/h in case of CVD-SLNs. The bioavailability of carvedilol enhanced by more than 2-folds when formulated as SLNs. This could be due to low aqueous solubility of carvedilol which negatively effect on the bioavailability of drug in suspension, while the incorporation of CVD in SLNs enhanced its solubility as well as its tissue permeability due to small nano-sized nature of SLNs. Additionally, the dependence of pharmacokinetic parameters of CVD upon delivery in the form of SLNs on the properties of the SLNs rather than by the physicochemical characteristics of the CVD molecule, for that, large proportion of the ingested SLNs usually absorbed into lymphatic circulation which bypass the first pass metabolism occur in liver.

**Table 3:** Pharmacokinetic parameters of carvedilol following oral administration of the optimized CVD-SLNs dispersion and the drug in suspension.

Pharmacokinetic parameter	CVD suspension	Optimized CVD-SLNs dispersion
C_max_ (ng/ ml)	51.52± 5.11	54.25 ± 6.91
t_max_ (h)	1.0 ± 0.5	4.0 ± 0.5
AUC_(0–24)_ (ng.h/ ml)	551.73± 69.12	844.21 ± 101.32
AUC_(24-∞)_ (ng.h/ ml)	34.89 ± 2.72	478.00 ± 23.15
AUC_(0-∞)_ (ng.h/ ml)	5*85*.12 ± 77.18	1322.21 ± 134.34
K_el_ (h^-1^)	0.122 ± 0.01	0.045 ± 0.02
t_1/2_ (h)	5.643 ± 0.63	15.31 ± 1.23
MRT (h)	8.737 ± 0.93	23.19 ± 1.33
CL (ml/h)	0.0042± 0.001	0.0021± 0.001
Relative bioavailability (%)	—	225.9

## 4. Conclusions

Short half-life, first pass metabolism, and low bioavailability due to poor solubility are barriers facing the management of hypertension and cardiac disease with carvedilol. In this research, carvedilol was reformulated as solid lipid nanoparticles and optimized utilizing Box-Behnken design in order to solve the previously mentioned problems associated with its use. The prepared SLNs prolonged the release and maintained the drug in plasma for more than 23 h and enhanced the oral bioavailability by more than 2-folds. Of course, it will not obviate the need for further clinical assessments for the prepared formulation on human to verify the results of experimental animals.

## Supporting information

S1 File In vitro release data. (XLSX)

S2 File Kinetic treatment of the release data. (RAR)
